# TTX-Resistant Sodium Channels Functionally Separate Silent From Polymodal C-nociceptors

**DOI:** 10.3389/fncel.2020.00013

**Published:** 2020-02-07

**Authors:** Robin Jonas, Vincenzo Prato, Stefan G. Lechner, Gerbrand Groen, Otilia Obreja, Fiona Werland, Roman Rukwied, Andreas Klusch, Marlen Petersen, Richard W. Carr, Martin Schmelz

**Affiliations:** ^1^Department of Experimental Pain Research, Medical Faculty Mannheim, University of Heidelberg, Mannheim, Germany; ^2^Institute of Pharmacology, University of Heidelberg, Heidelberg, Germany; ^3^Department of Anesthesiology, Groningen University, Groningen, Netherlands

**Keywords:** tetrodotoxin, activity-dependent slowing of conduction, axonal calcium imaging, action potential conduction, nociceptor classes

## Abstract

Pronounced activity-dependent slowing of conduction has been used to characterize mechano-insensitive, “silent” nociceptors and might be due to high expression of Na_V_1.8 and could, therefore, be characterized by their tetrodotoxin-resistance (TTX-r). Nociceptor-class specific differences in action potential characteristics were studied by: (i) *in vitro* calcium imaging in single porcine nerve growth factor (NGF)-responsive neurites; (ii) *in vivo* extracellular recordings in functionally identified porcine silent nociceptors; and (iii) *in vitro* patch-clamp recordings from murine silent nociceptors, genetically defined by nicotinic acetylcholine receptor subunit alpha-3 (CHRNA3) expression. Porcine TTX-r neurites (*n* = 26) *in vitro* had more than twice as high calcium transients per action potential as compared to TTX-s neurites (*n* = 18). In pig skin, silent nociceptors (*n* = 14) characterized by pronounced activity-dependent slowing of conduction were found to be TTX-r, whereas polymodal nociceptors were TTX-s (*n* = 12) and had only moderate slowing. Mechano-insensitive cold nociceptors were also TTX-r but showed less activity-dependent slowing than polymodal nociceptors. Action potentials in murine silent nociceptors differed from putative polymodal nociceptors by longer duration and higher peak amplitudes. Longer duration AP in silent murine nociceptors linked to increased sodium load would be compatible with a pronounced activity-dependent slowing in pig silent nociceptors and longer AP durations could be in line with increased calcium transients per action potential observed *in vitro* in TTX-resistant NGF responsive porcine neurites. Even though there is no direct link between slowing and TTX-resistant channels, the results indicate that axons of silent nociceptors not only differ in their receptive but also in their axonal properties.

## Introduction

Human (Serra et al., 1999; Weidner et al., 1999) and rat (Obreja et al., 2010; Serra et al., 2012; Petersson et al., 2014) C-nociceptors have been separated into a mechano-sensitive and mechano-insensitive (silent) class based on sensory and axonal characteristics. These two classes are in particular characterized by non-overlapping axonal properties: Activity-dependent slowing of conduction has been used to classify functional classes of nociceptors originally in the rat (Gee et al., 1996). It can be used to identify mechano-insensitive nociceptors in humans (Serra et al., 1999; Schmelz et al., 2000) and pig (Obreja et al., 2010). Higher expression of the voltage-sensitive sodium channel Na_V_1.8 in silent nociceptors leading to increased sodium influx (Petersson et al., 2014) and more pronounced slow inactivation (De Col et al., 2008) have been suggested to underlie the difference to polymodal nociceptors that only have moderate activity-dependent slowing of conduction. Silent nociceptors are of major clinical interest as their spontaneous activity has been linked to ongoing pain in chronic pain patients (Kleggetveit et al., 2012; Serra et al., 2015).

A molecular marker for silent nociceptors has been described in mouse, namely the nicotinic acetylcholine receptor subunit alpha-3 (CHRNA3; Prato et al., 2017), however, not yet in the human being. CHRNA3 positive neurons are mechano-insensitive peptidergic nociceptors mainly innervating deep somatic tissue and vice versa (Prato et al., 2017). Expression profiles of single primary afferents have been analyzed to classify them in an unbiased fashion into function classes (Usoskin et al., 2015; Zeisel et al., 2018). Indeed, high expression of CHRNA3 was found only in the peptidergic TrkA positive class of C-nociceptors (Usoskin et al., 2015), but we are facing a gap between molecular markers in rodents and functional classification in humans. The translation is problematic as major differences between mouse and human nociceptors have been reported concerning the overlap of Ret, TrkA and Na_V_1.8 expression (Rostock et al., 2018), but also the lack of correlation between small size DRG and nociceptive phenotype (Zhang et al., 2017). These differences impede direct translation between rodent and human or pig silent nociceptors.

In this study, we ask whether pronounced activity-dependent slowing in mechano-insensitive nociceptors is due to high expression of Na_V_1.8 and could, therefore, be characterized by their tetrodotoxin-resistance (TTX-r). High axonal expression of Na_V_1.8 should generate longer-lasting action potentials, thus leading to a more pronounced calcium influx. In order to test this hypothesis, we recorded intracellular calcium signals in single nerve growth factor (NGF)-dependent neurites from pig dorsal root ganglion cells using a modified compartmentalized Campenot chamber (Campenot et al., 2009; Pristerà et al., 2012; Klusch et al., 2013; Jonas et al., 2015). Porcine neurites were used because of the similarity of functional classes of DRG nociceptors between pig and human (Obreja et al., 2010). TTX-sensitivity was tested and compared to *in vivo* recordings of characterized single mechano-sensitive and mechano-insensitive nociceptors in the pig. In addition, we recorded from cold nociceptors which are known to express Na_V_1.8 (Zimmermann et al., 2007).

Traditionally, nociceptors have been classified based on their sensory profile whereas axonal conduction has been regarded as generic for C-fibers. However, it has been hypothesized that the interaction between specific transduction proteins with matching sets of voltage-sensitive axonal channels “will ultimately determine the characteristics of the propagated impulse discharge that encodes the properties of the stimulus, conferring functional specificity to the various types of sensory receptor neurons” (Belmonte and Viana, 2008). Data linking single-cell expression patterns of potassium channels in functionally defined primary afferents to their electrophysiological firing behavior support such a hypothesis (Chiu et al., 2014; Zheng et al., 2019). Using a known structural marker for silent nociceptors in the mouse and an established functional approach in the pig we identified neurons of this nociceptor class in the two species and searched for common axonal features that could contribute to their characteristic firing behavior and thereby might improve translation between species.

## Materials and Methods

### Animal Preparation

Ethical approval for experimental procedures was issued by the Ethics committee of the regional government (Karlsruhe, Baden-Wuerttemberg, Germany). Procedures followed the IASP guidelines for animal research and standard biosecurity and institutional safety procedures.

#### *In vitro* Preparation

Dorsal root ganglia (DRG) were removed post-mortem from 3 male piglets (*Sus scrofa domesticus*) ranging in age from P9 to P10. Piglets were initially sedated with intramuscular azaperone (Janssen-Cilag GmbH Neuss, Germany; 28 mg/kg) and ketamine (Essex Pharma GmbH, Munich, Germany; 70 mg/kg) and subsequently killed with a lethal dose of intracardial pentobarbital (20 mg/kg). The spine was removed, cleaned and stored in cold PBS (Sigma–Aldrich, Seelze, Germany). Following the mid-sagittal section, DRG were removed from all levels of the spinal cord and placed in DMEM (Sigma–Aldrich, Seelze, Germany).

#### *In vivo* Preparation

For surgery, pigs (*n* = 21; 16 male, five female) were pre-medicated as described previously (Obreja et al., 2010) with i.m. injection of azaperone (Stresnil^®^, Janssen Pharmaceutica, Beerse, Belgium) 2 mg/kg, atropine (Eifelfango^®^, Bad Neuenahr, Germany) 0.015 mg/kg and midazolam 1 mg/kg. General anesthesia was induced with Propofol^®^ (Fresenius, Bad Homburg, Germany) 2 mg/kg i.v., and maintained with pentobarbital (Narcoren^®^, Merial, Halbergmoos, Germany) 8–14 mg/kg/h. Pigs were intubated, ventilated and vital parameters (heart rate, O_2_ saturation, rectal temperature) were monitored.

### Cell Culture of Pig DRG Neurons

Isolation and culture of pig DRG neurons were similar to previously described procedures (Petersen et al., 1996; Obreja et al., 2008; Jonas et al., 2015). Briefly, DRG was freed mechanically from connective tissue and incubated at 37°C for 110 min in DMEM containing Gentamicin and collagenase (Invitrogen, Life Technologies, Schwerte, Germany) during which time half of the medium was replaced with fresh medium twice. Ganglia were rinsed twice in PBS devoid of Ca^2+^ and Mg^2+^ and incubated for 8 min at 37°C in trypsin (Sigma–Aldrich, Seelze, Germany). Ganglia were placed in a mixture of DMEM and Ham’s F-12 (Gibco, Life Technologies, Schwerte, Germany) and triturated with a fire-polished siliconized Pasteur pipette to isolate somata. Cells were subsequently transferred to 10% Percoll solution and centrifuged (740 RZB, 7 min) to remove connective tissue. The resulting pellet was twice washed in DMEM and centrifuged (170 RZB, 5 min).

Cells were cultured in the central compartment of a three-compartment Campenot chamber (Campenot et al., 2009) in Ham’s F12 medium supplemented with 10% heat-inactivated horse serum (Gibco, Life Technologies, Schwerte, Germany), 2 mM L-glutamine, 100 U/ml penicillin and 10 μg/ml streptomycin. The medium was supplemented with rhß-NGF (Calbiochem, Schwalbach, Germany), 50 ng/ml in the central and 100 ng/ml in the lateral compartments. Cells were kept in the culture at 37°C in a 5% CO_2_ humidified atmosphere and half of the medium was replaced every 2–3 days. Experiments were performed after 4–8 days in culture when neurites had grown from the center into the lateral compartments (Klusch et al., 2013, 2018).

### Calcium Imaging Setup

In preparation for calcium imaging, the culture medium was removed from all three compartments of the Campenot chamber and neurons were washed three times with imaging buffer (in mM: 140 NaCl, 2 KCl, 2 CaCl_2_ × 6 H_2_O, 1 MgCl_2_ × 6 H_2_O, 20 d-glucose, 10 HEPES, pH 7.4). After washing, the intensity-based calcium indicator Fluo-8^®^, AM (AAT Bioquest, Sunnyvale, CA, USA), diluted in imaging buffer to a concentration of 2 μM was added into all compartments. Cells were incubated with fluorescent dye for 30 min at room temperature before being washed three times with imaging buffer and perfused thereafter with imaging buffer at a flow rate of 3 ml/min (Minipuls^®^3, Gilson, Middleton, WI, USA) in a darkened room. Fluorescence and brightfield images were acquired using a back-illuminated 512 * 512 pixel cooled EMCCD camera (Evolve 512, Photometrics, Tucson, AZ, USA). The camera was connected to the side port of an inverted microscope (Axiovert 200, Zeiss, Jena, Germany). Bright-field illumination was provided by the microscope’s halogen lamp. A 465 nm LED (Prior Scientific, Rockland, MA, USA) and a filter set (excitation BP 450–490 nm, dichroic = 510 nm, emission = 515 nm LP, Chroma Technologies) were used for excitation and emission of Fluo-8^®^, AM. Fluorescence images were acquired using μManager (Edelstein et al., 2010) software with electrical stimulation and image acquisition synchronized *via* an Arduino Duemilanove (Watterott electronic, Leinefelde, Germany). Fluorescence image time sequences were recorded at 20 Hz before and during the electrical stimulation period.

### Imaging Data Analysis

**Figure 1:**
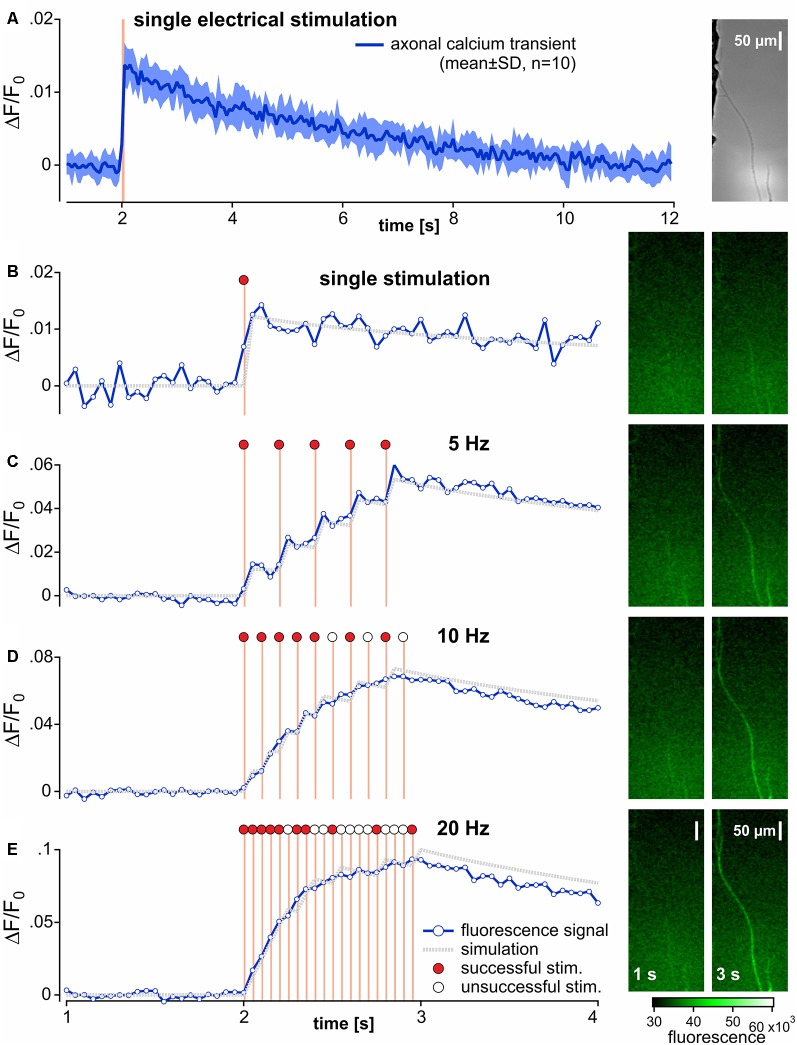
Recording and analysis of calcium transients in outgrowing neurites from pig dorsal root ganglia neurons. **(A)** Specimen of the axonal calcium transient (Fluo-8, AM) in a single neurite induced by one electrical stimulation (orange bar) recorded at 20 Hz for 12 s with 10 repetitions (brightfield image of the specimen in right panel). The averaged axonal calcium transients were fitted to a double-exponential function and used as impulse-response (gray dotted trace) for single responses **(B)**. Corresponding specimens of the fluorescence signal before and at end of electrical stimulation are shown on the right panel of each stimulation frequency **(B–E)**. Axonal calcium transients to 1 s bursts of stimuli at 5, 10 and 20 Hz stimulus frequencies are shown in panels **(C–E)**. In an attempt to evaluate the number of successful electrical stimulations evoking an axonal calcium gradient, the impulse-response derived from **(A)** was used. In this simulation (gray dotted lines), the input signal was set to 0 (open circles) for unsuccessful stimulation or to 1 (red circles) for successfully adapted to ideally match to the original data. Note, that adequate approximations were achieved for 5 Hz stimulation, but not for 10 and 20 Hz.

Image analysis was performed using ImageJ-software (NIH). Regions of interest (ROI) were delineated by drawing a line along the entire length of the visible neurite as seen in the bright-field image (Figure 1A, right panel). The time profile of fluorescence intensity for each pixel along this linear ROI was determined from each image using the Reslice function in ImageJ. The resulting data array was processed using custom-written routines in Igor Pro (WaveMetrics, Lake Oswego, OR, USA).

Fluorescence intensity (F) signals were averaged across all pixels of the ROI at each time point. The resulting time series was corrected for bleaching in the following manner. Baseline fluorescence (F_0_) was determined by a single exponential function including 20 control values before electrical stimulation and the last 20 values of the fluorescence time series. The difference between averaged fluorescence intensity (F) and baseline fluorescence values (F_0_) was calculated (ΔF) and divided by the fluorescence fit value at each time point to generate a normalized fluorescence ratio (ΔF/F_0_). To quantify calcium responses to electrical stimulation, the maximum positive peak and area under the curve (AUC) were calculated from the ΔF/F_0_ time series.

Positive calcium responses were defined as a significant increase of ΔF/F_0_ between baseline and stimulus value comparing fluorescence ratios during 1 s before and during the stimulation period using an unpaired *T*-test.

The impulse-response function for electrical stimulation was determined before and after exposure to TTX in each neurite. ΔF/F_0_ was averaged from 10 subsequent single stimulations (Figure 1A). A double-exponential function starting from the initiation of this pulse was then calculated from averaged ΔF/F_0_. To correct for any possible decreases in fluorescence between the 10 repetitive stimulations, AUCs were normalized to the level of the 10th stimulation (Figure 1B). The resulting function was defined as the impulse calcium response (Figure 1B, gray dotted trace). Action potential conduction was indirectly assessed by measuring electrically evoked calcium signals. To estimate the number of electrically induced calcium transients, a predicted calcium response profile was determined by the convolution of the empirical impulse calcium response to the electrical stimuli. Each individual pulse interval during the stimulation period was iteratively analyzed for the difference between empirical and computed ΔF/F_0_ values. According to the closest match of empirical and computed data, the response was considered to be either 1 (successful) or 0 (non-successful). I. e., if the computed data with an impulse signal = 1 returned a smaller difference to the empirical data than the computed data with an impulse signal = 0, electrical stimulation was considered to have induced a calcium response and vice versa.

### *In vitro* Experimental Protocols

#### Control Electrical Stimulation

Neurites were first identified under bright-field illumination in the two lateral compartments of the chamber (Figure 1A, right panel). Initially, neuronal vitality was determined by monitoring the calcium response to electrical stimulation. Constant current field stimulation (20 pulses at 20 Hz, 40 mA, 1 ms pulse width) was applied in the central compartment (Digitimer DS7A, Letchworth Garden City, UK) in order to incite action potentials that were conducted along the neurites into the lateral chamber (Jonas et al., 2015). Neurites that did not respond to electrical stimulation with an increase in fluorescence were discarded.

#### Determination of the Impulse-Response Function

Neuronal ΔF/F_0_ was measured during 10 repetitive fluorescence responses at intervals of at least 2 min. Changes in neuronal fluorescence intensity were evoked by a time-locked single pulse and data were further processed by a custom-written routine in Igor Pro (WaveMetrics, Lake Oswego, OR, USA) to determine individual impulse-response functions. See “Imaging Data Analysis” section for further details.

#### Frequency Dependence of Electrically Evoked Calcium Fluorescence Transients

To examine fluorescence intensity as a function of electrical stimulation rate, the axonal responses to 1 s bouts of electrical stimuli comprising 1, 5, 10 and 20 pulses were recorded at intervals of not less than 120 s.

#### Sodium Channel Blocker

After the initial determination of the impulse-response function and frequency dependence of electrically evoked calcium fluorescence, TTX (500 nM) was added to the perfusate for the lateral compartment. After 10 min of perfusion time, impulse-response function and frequency dependence of electrically evoked calcium fluorescence transients were determined in TTX-exposed neurites.

### *In vivo* Experimental Protocols

#### Extracellular Single Fiber Recordings

As described previously (Obreja et al., 2010), saphenous nerves were exposed at mid-thigh over a length of about 6 cm and the teased fiber technique (Campbell and Meyer, 1983; Gee et al., 1999) was used to extracellularly record action potentials from single nerve fibers. Signals were amplified (Model 5113, Ametek Inc., TN, USA), audio monitored, filtered (Model 3364, Krohn-Hite Corporation, Brockton, MA, USA) and displayed on an oscilloscope. Characteristic C-fiber discharge upon squeezing the skin in the innervation territory of the saphenous nerve guided the electrical search strategy used to identify individual C-units. Electrical stimuli (20 mA; 0.5 ms) were generated at 0.25 Hz by a constant current stimulator (DS7A, Digitimer Limited, Hertfordshire, UK) and applied to the skin through two non-insulated microneurography electrodes (FHC Inc., Bowdoin, ME, USA). Needles were inserted intradermally at sites where time-locked, electrically evoked action potentials with long latencies (~100–200 ms) could be elicited. The current intensity was adjusted at 1.5-times the electrical threshold. The shortest distance between the stimulation needles and the recording electrode was measured and divided by the latency recorded after a 2-min pause to calculate the resting conduction velocity (CV). All fibers in this study had CV values <2 m/s. Action potentials were amplified, processed online, and displayed on a computer using DAPSYS 8.0, a joint hardware and software system designed for real-time acquisition, window discrimination and latency measurements of the action potentials (for technical details, see Turnquist et al., 2004,^1^).

#### Characterization of C-fiber Classes

Single C-fibers were classified according to their responses to mechanical stimulation (brush, v.Frey filaments 10–600 mN) and cold application (ice cube for 60 s) into three classes: mechano-sensitive (CM) nociceptors (brush negative, mechanical threshold between 10 and 150 mN), mechano-insensitive (CMi) nociceptors (mechanically insensitive) and cold (CN) nociceptors (mechano-insensitive, activation by noxious cold; Obreja et al., 2010). Slowing of conduction to repetitive electrical stimulation at 2 Hz for 3 min was assessed after a 2-min pause. Moreover, we applied three trains of 25 electrical pulses at 5 Hz with an interval of 10 s and counted the number of action potentials induced. For silent nociceptors, the frequency was reduced to 1 Hz, if they did not follow the 5 Hz such that all fibers responded to >90% of the pulses before the TTX.

#### TTX-Injections

One-hundred microliters of TTX dissolved in PBS was injected intracutaneously within the receptive field of the nociceptor, the injection bleb covering the two non-insulated intracutaneous stimulation electrodes. Electrical stimulation at 0.25 Hz was given during the injection to verify the activation and conduction of the unit. Stimulation intensity was adjusted to compensate for possible threshold changes upon TTX up to a stimulation intensity of 100 mA. Five minutes after the injection, the electrical threshold was measured and the stimulation (three trains, 25 pulses each, 5 Hz) was repeated at 1.5-fold activation threshold. Again, the number of recorded action potentials was measured and normalized to the baseline level prior to TTX, which was injected at increasing concentrations of 10 and 100 nM, 1 and 10 μM. If the electrical pulses still induced action potentials after TTX, injections of higher TTX concentrations were performed.

### Chemicals

All chemicals were obtained from commercial sources. TTX citrate (Tocris, UK) was prepared in stock solutions of PBS and distilled water. Stock solutions were aliquoted and stored frozen before being diluted to the desired concentration in imaging buffer or PBS (for extracellular single fiber recordings) on the day of the experiment.

#### Culture of Murine DRG Somata

Mouse DRG primary cultures were prepared from 12 to 18 weeks old CHRNA^+^ mice [official name Tg(Chrna3-EGFP)BZ135Gsat/Mmnc (RRID:MMRRC_000243-UNC)] of both sexes that were killed by placing them in a CO_2_-filled chamber for 2–4 min followed by cervical dislocation. L2-L5 DRGs were collected in Ca^2+^ and Mg^2+^-free PBS and incubated in a mixture of Collagenase type I (2.0 μg/ml, Sigma) and Trypsin (1.5 μg/ml, Sigma) for 60 min at 37°C. Digested DRG’s were washed twice with growth medium [DMEM-F12 (Invitrogen) supplemented with L-glutamine (2 μM, Sigma), glucose (8 mg/ml, Sigma), penicillin (200 U/ml)—streptomycin (200 μg/ml; both Life Technologies) and 5% fetal horse serum (Life Technologies)]. Ganglia were triturated with a fire-polished Pasteur pipette and the suspension plated in a droplet of growth medium on glass coverslips pre-coated with poly-L-lysine (20 μg/cm^2^, Sigma) and laminin (4 μg/cm^2^, Life Technologies). To allow neurons to adhere, the coverslips were kept for 3–4 h at 37°C in a humidified 5% incubator before being flooded with fresh growth medium.

#### Patch-Clamp Protocol

Cultures were used for patch-clamp experiments the next day. Whole-cell patch-clamp recordings were made at room temperature (20–24°C). Patch pipettes with a tip resistance of 2–4 MΩ were pulled (Flaming-Brown puller, Sutter Instruments, Novato, CA, USA) from borosilicate glass capillaries (BF150-86-10, Sutter Instrument), filled with a solution consisting of 110 mM KCl, 10 mM NaCl, 1 mM MgCl_2_, 1 mM EGTA, 10 mM HEPES, 2 mM guanosine 5′-triphosphate (GTP) and 2 mM adenosine 5′-triphosphate (ATP) adjusted to pH 7.3 with KOH. The bathing solution contained 140 mM NaCl, 4 mM KCl, 2 mM CaCl_2_, 1 mM MgCl_2_, 4 mM glucose, 10 mM HEPES, adjusted to pH 7.4 with NaOH. The RMP slightly varied between cells and hence all cells were set to a holding level of −60 mV. All recordings were made in current-clamp mode using an EPC-10 amplifier (HEKA, Lambrecht, Germany) in combination with Patchmaster^©^ and Fitmaster^©^ software (HEKA). Pipette and membrane capacitance was compensated using the auto function of Patchmaster. Action potentials were evoked in current-clamp mode by injecting a series of incrementing (Δ40 pA) rectangular current pulses of 200 ms duration. The first action potential that was elicited by this approach was used for the analysis.

### Statistics

Statistical tests were performed in STATISTICA 7.1 (StatSoft Inc, Tulsa, OK, USA). Peak ΔF/F_0_, AUC and neuronal conduction between both categorical groups of conducting neurites (TTX-R vs. TTX-S) were compared by two-way ANOVA. Repeated measures ANOVA was used for within-group comparison of peak ΔF/F_0_, AUC and neuronal conduction in TTX-R neurites before and after TTX exposure. Bonferroni-corrected *t*-tests were used for *post hoc* analysis. Student’s *t*-test was used to compare peak ΔF/F_0_ and AUC of the impulse-response between TTX-R and TTX-S conducting neurites (unpaired *t*-test) and within TTX-R neurites before and after TTX exposure (paired *t*-test). Linear correlation of individual peak ΔF/F_0_ of TTX-R neurites before and after TTX exposure was determined by Pearson’s r. Group data are presented as mean ± standard deviation (SD) or arithmetic mean from averaged data ± standard error of the mean (SEM). The level of statistical significance is indicated in the figures as either * for *p* < 0.05 or ** for *p* < 0.01.

## Results

### *In vitro* Recordings

**Figure 2:**
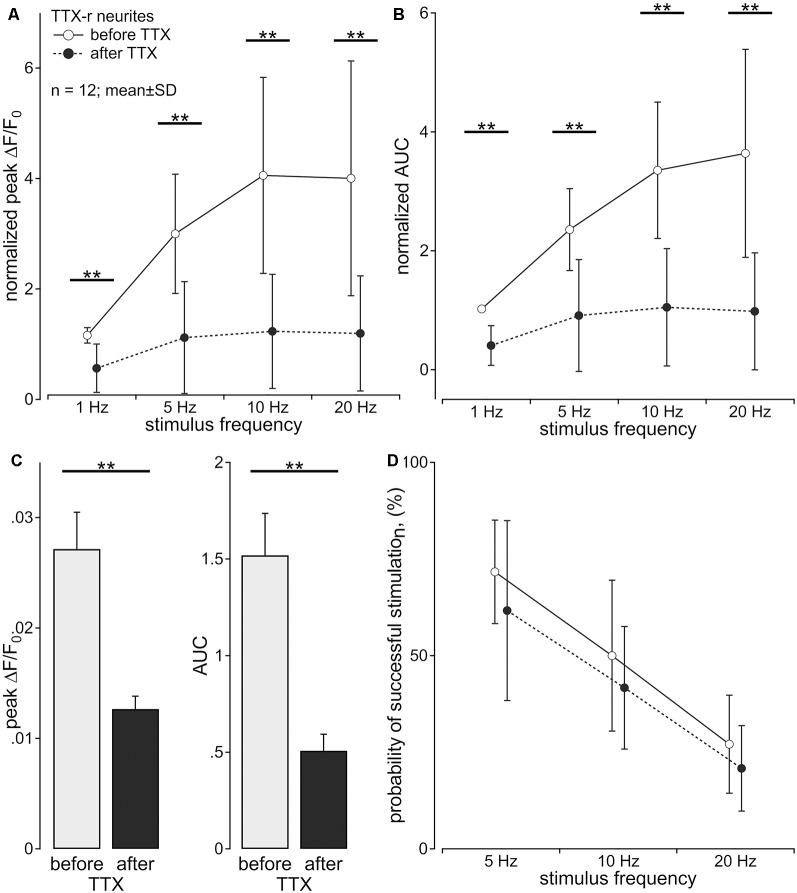
Axonal calcium transients are reduced by tetrodotoxin (TTX). Calcium transients of TTX-resistant neurites (*n* = 12; pig) following to sequential stimulation with 1, 5, 10 and 20 pulses over 1 s were assessed before and during exposure to TTX (500 nM) in the same neuron. **(A)** Normalized peak calcium transient (ΔF/F_0_ ; main effect, *F*_(1,11)_ = 53.45, **p < 0.001) and **(B)** normalized area under the curve (AUC; main effect, *F*_(1,11)_ = 83.88, **p < 0.001) were reduced by TTX for all stimulus frequencies. **(C)** Non-normalized calcium transients in response to single electrical stimulation showed the same reduction after TTX [peak: 0.027 ± 0.011 standard deviation (SD) vs. 0.013 ± 0.004; *t*_(11)_ = 5.0, **p < 0.001, paired *t*-test; **C**, left panel; AUC: 1.519 ± 0.753 (SD) vs. 0.507 ± 0.298; *t*_(11)_ = 5.3, **p < 0.001, paired *t*-test; **C**, right panel)]. **(D)** Probability of successful electrical stimulation declined with stimulation frequency. No significant difference of this probability was observed before and after TTX exposure (main effect, *F*_(1,11)_ = 1.99, *p* > 0.05, repeated measures ANOVA).

The multiple compartment configuration of the culture chamber allowed us to electrically stimulate the somata in the central compartment and record calcium transients in neurites in the lateral compartment (Jonas et al., 2015). We recorded calcium signals from 64 individual outgrowing neurites; 53 of these responded to a single electrical pulse and were tested further. Of these, 43 neurites survived the entire stimulation protocol and were analyzed. Neurites were deemed TTX-r, when a positive calcium signal was evident in response to 20 Hz electrical stimulation (*n* = 26) in the presence of TTX (500 nM). TTX sensitive (TTX-s) neurites failed to respond (*n* = 17). In 12 (TTX-r) neurites calcium responses were recorded for all stimulus frequencies (5–20 Hz) both before and during TTX (Figure 2). TTX application in these 12 neurites reduced the intensity of the calcium transients to less than 50% for trains of stimulation up to 20 Hz during the stimulation period (normalized peak: main effect, *F*_(1,11)_ = 53.45, *p* < 0.001); Figure 2A; normalized AUC: main effect, *F*_(1,11)_ = 83.88, *p* < 0.001; Figure 2B) with peak and AUC for single pulses also dropping by more than 50% [peak: 0.027 ± 0.011 (SD) vs. 0.013 ± 0.004; *t*_(11)_ = 5.0, *p* < 0.001, paired *t*-test; Figure 2C, left panel; AUC (1.519 ± 0.753 (SD) vs. 0.507 ± 0.298; *t*_(11)_ = 5.3, *p* < 0.001, paired *t*-test; Figure 2C, right panel)]. However, when estimating the number of conducted action potentials based on the calcium transients, TTX did not significantly reduce the number of action potentials expected based on the impulse-response relation (see Figure 1) even at stimulation frequencies up to 20 Hz (main effect, *F*_(1,11)_ = 1.99, *p* = 0.19; Figure 2D).

### Calcium Influx Per Action Potential Is Increased in TTX-r vs. TTX-s Neurites

**Figure 3:**
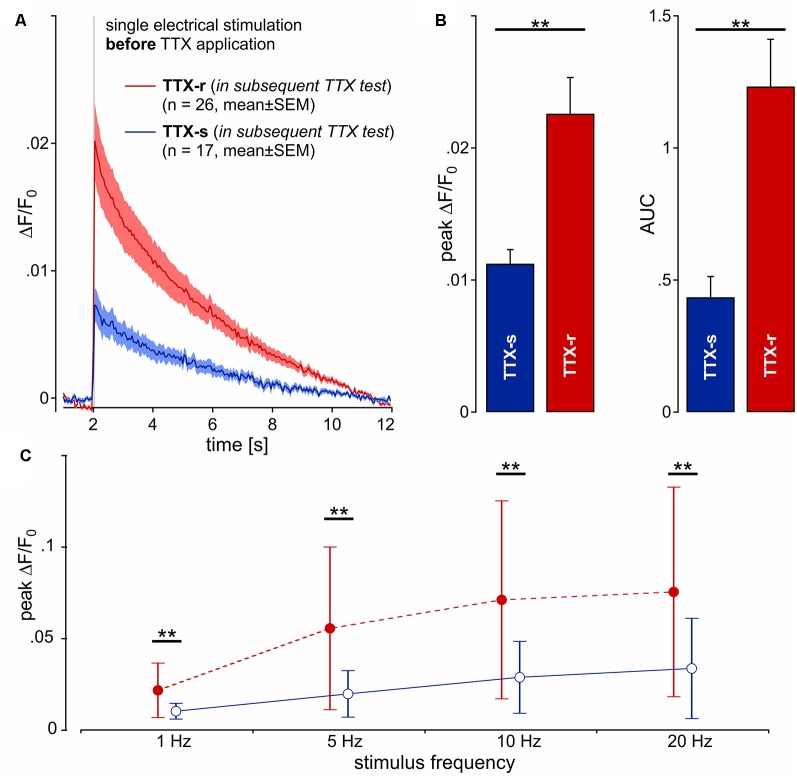
Calcium transients (ΔF/F_0_) from 43 neurites (pig) in response to one single electrical pulse (40 mA, 1 ms) were averaged from 10 repetitions each. **(A)** Kinetics of the calcium transients *before* TTX exposure are shown for two groups of neurites: one that was shown *later* to be blocked by TTX (TTX-s, *n* = 17; blue line) and a second that was still conducting (TTX-r, *n* = 26; red). **(B)** Peak calcium transient (ΔF/F_0_) and AUC was increased in TTX-resistant (TTX-r) neurites as compared to TTX-s conducting neurites (peak and AUC, respectively: *t*_(41)_ = 3.2, **p < 0.01 and *t*_(41)_ = 3.47, **p < 0.01, unpaired *t*-test). **(C)** Averaged data from peak calcium responses during stimulation in response to stimulation with 1, 5, 10 and 20 pulses over 1 s. TTX-r calcium responses (red) were higher than TTX-s calcium responses (blue) for all stimulus frequencies in peak ΔF/F_0_ (main effect, *F*_(1,41)_ = 9.6, **p < 0.01, repeated measures ANOVA).

After the application of TTX, 26 neurites were still conducting (TTX-r), whereas 17 neurites were completely blocked (TTX-s). Unexpectedly, when comparing the initial calcium transients of neurites evoked by electrical stimulation before TTX application between those neurites that later were found to be TTX-r and those that were later completely blocked by TTX, we observed a striking difference: the amplitude of calcium transients to single stimulation (Figure 3A) was significantly higher in TTX-r neurites by a factor of more than 2 [0.023 ± 0.014 (SD) vs. 0.011 ± 0.004; *t*_(41)_ = 3.24, *p* = 0.002, unpaired *t*-test; Figure 3B, left panel]. Accordingly, AUC values were increased in TTX-r neurites [1.233 ± 0.91 (SD) vs. 0.435 ± 0.324; *t*_(41)_ = 3.47, *p* = 0.001, unpaired *t*-test; Figure 3B, right panel]. Also for bursts of electrical stimuli at 5, 10 and 20 Hz for 1 s, peak ΔF/F_0_ (main effect, *F*_(1,41)_ = 9.6, *p* = 0.004, repeated measures ANOVA; Figure 3C) and AUC (main effect, *F*_(1,41)_ = 10.77, *p* = 0.002, repeated measures ANOVA; data not shown) were increased at all stimulus frequencies consistent with results from single pulses. Differences in calcium-responses between TTX-r and TTX-s neurons and before vs. after TTX-application are summarized in Supplementary Table S1.

### *In vivo* Recordings: TTX Sensitivity of Different Nociceptor Classes in the Pig

**Figure 4:**
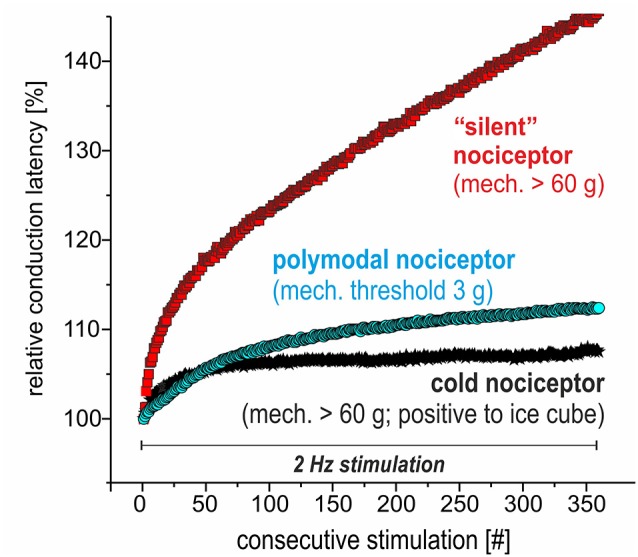
Specimen of extracellular *in vivo* recordings from single nociceptors in the saphenous nerve in pig visualizing the electrical stimulation protocol to assess activity-dependent slowing of conduction velocity (CV). Three different C-nociceptors [polymodal (light blue), mechano-insensitive (red) and cold nociceptors (dark blue)] are stimulated electrically inside their innervation territory in the leg and recorded in the proximal saphenous nerve. The electrical stimulation protocol consists of 360 pulses at 2 Hz for 3 min. Note that the activity-dependent slowing is extreme for mechano-insensitive “silent” nociceptors and moderate for polymodal nociceptors. Cold nociceptors are mechano-insensitive but are characterized by an even less pronounced activity-dependent slowing as compared to polymodal nociceptors.

We recorded from 12 polymodal nociceptors (CV 1.08 ± 0.2 m/s; mean ± SD), 14 mechano-insensitive “silent” nociceptors (CV 0.95 ± 0.18 m/s) and five cold nociceptors (CV 1.58 ± 0.14 m/s). The magnitude of activity-dependent slowing of conduction in response to 3 min of 2 Hz stimulation was lowest in the cold nociceptors (8.3 ± 2.2%) followed by polymodal (12.2 ± 1.6%) and highest in mechano-insensitive nociceptors (31.5 ± 13%) as expected (specimen in Figure 4).

**Figure 5:**
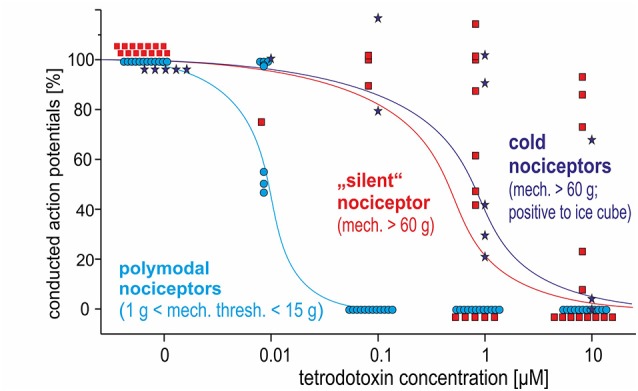
Dose-response curves for TTX-induced block of action potential generation and conduction in three different classes of C-nociceptors in pig, *in vivo*: polymodal (light blue), mechano-insensitive (“silent,” red) and cold nociceptors (dark blue stars). While all polymodal nociceptors are blocked following intracutaneous injection of TTX at 100 nM, silent nociceptors and cold nociceptors still conduct even after TTX-injections at 1 μM.

Polymodal nociceptors showed the highest TTX sensitivity with all of them being completely blocked after an intradermal injection of TTX at a concentration of 100 nM. Both, mechano-insensitive and cold nociceptors had a significantly lower TTX sensitivity as none of these fibers was blocked after injection of 100 nM TTX. Moreover, some of these fibers were still generating and conducting action potentials in the presence of TTX at 1 μM or even 10 μM (Figure 5).

### Action Potential Recordings in Murine Silent Nociceptors

**Figure 6:**
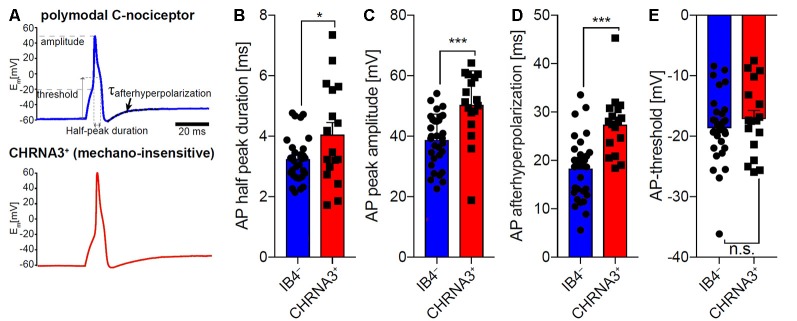
Comparison of the action potential properties of silent nociceptors and polymodal C-nociceptors in mice. **(A)** Example traces of action potentials recorded from small diameter isolectin B4 (IB4) negative/capsaicin-sensitive (i.e., polymodal C-fiber nociceptors, top trace) and nicotinic acetylcholine receptor subunit alpha-3 positive (CHRNA3^+^) silent nociceptors (bottom trace). Action potential (AP) half-peak duration **(B)**, AP peak amplitude **(C)** and recovery from hyperpolarization (τ_AHP;_ time constant of after-hyperpolarization) **(D)** significantly differed between the two populations, whereas AP thresholds **(E)** were indistinguishable. Bars represent means ± standard error of the mean (SEM) and were compared by Student’s *T*-test. *p < 0.05, ***p < 0.001.

To compare the properties of action potentials of mechano-insensitive nociceptors and polymodal C-fiber nociceptors in mice, we utilized a reporter mouse line in which mechano-insensitive nociceptors that can readily be identified in primary DRG cultures by means of EGFP expression (Prato et al., 2017; Schaefer et al., 2018). Polymodal C-fiber nociceptors were identified as small-diameter neurons that are not labeled by the isolectin-B4 (IB4) and respond to the TRPV1 agonist capsaicin (applied to the cells after recording action potentials). Action potentials of CHRNA3^+^ silent nociceptors and IB4^−^ polymodal C-fiber nociceptors had similar activation thresholds, but differed in several other respects (Figure 6). Thus, the action potential duration measured at 50% peak amplitude was significantly longer in CHRNA3^+^ neurons (4.05 ± 0.39 ms) than in IB4^−^ neurons (3.24 ± 0.13 ms; Figures 6A,B). Moreover, the peak amplitudes of the action potentials significantly differed between the two populations (IB4^−^; 38.76 ± 1.53 mV vs. CHRNA3^+^ 50.35 ± 2.73 mV; Figure 6C). To test if the action potentials in the two populations also differ with respect to hyperpolarization, we fitted the recovery from hyperpolarization with a single exponential equation and compared the time constants of recovery from after-hyperpolarization (τ_AHP_, Figure 6D). This analysis showed that action potentials in CHRNA3^+^ neurons recover much slower from hyperpolarization than those recorded from IB4^−^ neurons (IB4^−^; τ_AHP_ = 18.37 ± 1.18 ms vs. CHRNA3^+^: τ_AHP_ = 27.38 ± 1.54 ms, Figure 6D).

## Discussion

Using a compartmentalized DRG neuron culture system of single neurites, we successfully quantified axonal calcium gradients accompanying the conduction of single action potentials. The calcium gradient induced by a single action potential under control conditions was about twice as high in those neurites later shown to be TTX-resistant as compared to TTX-sensitive neurites. *in vivo* recordings in the pig identified mechano-insensitive, “silent” nociceptors and cold nociceptors to be particularly resistant to TTX (>1 μM), whereas conduction in polymodal nociceptors was blocked by TTX at concentrations of 0.1 μM. In the absence of a marker of silent nociceptors in pig or human, we compared our data to electrophysiological characteristics of CHRA3+ murine silent nociceptors and small diameter IB4^−^ nociceptors, i.e., putative polymodal C-fiber nociceptors. Single action potentials in silent nociceptors had a longer duration, higher amplitude and longer-lasting after-hyperpolarization all of which would be in line with increased calcium- and sodium influx per action potential and increased activity-dependent slowing. Thus, our *in vitro* data showing an increased calcium gradient in TTX-r axons in response to electrical stimulation might also correlate to increased sodium influx and thus appears to be in line with higher TTX-resistance and more pronounced activity-dependent slowing in silent nociceptors. However, also cold nociceptors were found to be TTX-r, but they are characterized by a particularly low activity-dependent slowing of conduction (Obreja et al., 2010). Thus, high expression of TTX-r sodium channels alone is not sufficient to explain activity-dependent slowing by increased sodium influx per action potential.

### Discharge Frequency (Pig, *in vitro*)

Calcium imaging of single neurites revealed that fidelity of action potential conductions fell from about 100% at 1 Hz to about 30% at a stimulation frequency of 20 Hz. In general, discharge frequencies of C-nociceptors are expected in the range of about 20–80 Hz for few impulses, but they can reach up to 180 Hz for short bursts (Weidner et al., 2002). On the other hand, longer-lasting stimulation, such as 2 Hz for 3 min often leads to blocking of mechano-insensitive nociceptors (Obreja et al., 2011). However, it also needs to be considered that our *in vitro* measurements were assessed at room temperature, and therefore we might underestimate maximum discharge frequencies at skin temperatures of about 34°C under *in vivo* conditions.

### TTX Effects (Pig, *in vitro*)

About 60% of the neurites were still conducting action potentials after TTX (500 nM). This percentage is relatively high when considering compound action potential data of peripheral nerves showing a 90% reduction in compound action potential amplitude following TTX (500 nM). However, recent data suggest that TTX sensitivity is lower when distal nerves are investigated: thus, TTX-sensitivity was significantly lower in distal sural and peroneal nerve in mouse and monkey (Klein et al., 2017). These results are also in line with observations that spike initiation of corneal nociceptors was found TTX resistant, whereas action potential conduction in the ciliary nerve was blocked by TTX (Brock et al., 1998). Interestingly, no major drop in response rate to electrical pulses was observed after TTX, but calcium influx was reduced by more than 50%. Thus, albeit conduction was preserved, the calcium transient per action potential was reduced, at least for short bursts at 20 Hz suggesting that the changed shape of the action potential reduced voltage-dependent calcium currents. Considering that the duration of single action potentials is expected to be longer after blocking TTX–s channels, one might have even expected increased calcium transients for broader action potentials as observed for central (Borst and Sakmann, 1999; Geiger and Jonas, 2000) neurons. On the other hand, a more shallow slope and reduced amplitude of the action potential after TTX could explain reduction of the calcium gradient as shown in dorsal root ganglion neurons (Park and Dunlap, 1998). While calcium gradients per action potential were reduced after TTX, the numbers of electrically induced action potentials suggested that TTX-r currents were still sufficient for regular action potential conduction. However, this result cannot be directly related to characteristics of silent vs. mechanosensitive nociceptors as dissection and culture conditions are expected to change the phenotype of the primary afferents.

### TTX Effects (Pig, *in vivo*)

Our *in vivo* data revealed that mechano-insensitive and cold nociceptors have TTX-r action potentials, whereas polymodal nociceptors were TTX sensitive. It is important to note that TTX-r currents are facilitated by colder temperature (such as in our *in vitro* experiments at room temperature), “unmasking” action potentials by cooling (Zimmermann et al., 2007). Therefore, in the *in vivo* situation at around 34°C dose-response curves for TTX will be shifted to the right leading to lower TTX sensitivity of the silent and cold nociceptors as compared to the *in vitro* condition. Yet, there was still a distinct difference to the polymodal nociceptors that were blocked at 0.1 μM TTX. High expression of Na_V_1.8 on cold nociceptors is not surprising considering the lack of cold pain in Na_V_1.8 deficient mice (Zimmermann et al., 2007). There has been no direct evidence for TTX-r conduction in silent nociceptors, but selective enhancement of slow inactivation by lacosamide preferentially blocked their conduction (Obreja et al., 2012). However, high expression of Na_V_1.8 in mechano-insensitive nociceptors has been suggested before based on long-lasting discharge under depolarized conditions such as experimental capsaicin application (Schmelz et al., 2000; Wooten et al., 2014) or supposedly depolarized conditions in chronic pain patients (Kleggetveit et al., 2012). Also, the characteristic pronounced activity-dependent slowing of conduction in silent nociceptors (Serra et al., 1999; Weidner et al., 1999) has been linked to increased sodium influx *via* Na_V_1.8 based on modeling results (Petersson et al., 2014; Tigerholm et al., 2014). This suggestion was supported recently in a patient with Na_V_1.8 mutation who revealed increased activity-dependent slowing patterns in silent nociceptors (Kist et al., 2016).

### Nociceptor Class-Specific Activity-Dependent Slowing of Conduction (Pig, *in vivo*)

The pronounced activity-dependent slowing of conduction has been used as characteristic criterion to differentiate axons of silent nociceptors from those of polymodal nociceptors (Serra et al., 1999; Weidner et al., 1999) and increased sodium influx per action potential *via* TTX-r Na_V_1.8 in silent nociceptors has been assumed as underlying mechanism (Petersson et al., 2014). We found increased calcium transients per action potential in TTX-r neurites that might be linked to enhanced activity-dependent slowing observed in the TTX-r silent nociceptors. However, activity-dependent slowing of cold nociceptors that were also shown to be TTX-r is even lower than in TTX-s polymodal nociceptors. Thus, even though high sodium currents and propensity to slow inactivation favors the role of Na_V_1.8 for the induction of activity-dependent slowing, it is not sufficient to determine the degree of slowing. In particular, higher Na^+^/K^+^ pump activity and decreased expression of the delayed rectifier potassium channel have been shown in modeling experiments to reduce activity-dependent slowing (Petersson et al., 2014) and could explain reduced activity-dependent slowing in cold nociceptors. Moreover, sodium and calcium influx during action potentials is differentially regulated (Bean, 2007). In particular, short-lasting action potentials that might be found in cold-sensitive primary afferents (Djouhri et al., 1998) will limit sodium influx but may favor calcium entry (Park and Dunlap, 1998). Moreover, increased intracellular calcium will also secondarily gate ion channels that contribute to activity-dependent excitability changes such as calcium-activated potassium channels (e.g., sK1; Boettger et al., 2002) and calcium-activated chloride channels (e.g., TMEM16A; Ru et al., 2017). Independent of accumulation of intracellular sodium, a hyperpolarized membrane potential and reduced length of the terminal branch could reduce activity-dependent slowing (Petersson et al., 2014) in cold nociceptors. Thus, the combination of TTX-resistance and reduced slowing of conduction in cold nociceptors could be explained by higher Na^+^/K^+^ pump activity, fewer delayed rectifier potassium channels, hyperpolarized membrane potential or shorter terminal branches.

### Comparison to Rodents

The lack of molecular markers for silent nociceptors in human and pig precludes direct investigations in *ex vivo* and *in vitro* systems. In rodents, intracellular *in vivo* recordings of functionally defined nociceptors have been used (Djouhri et al., 2003; Acosta et al., 2014) to bridge that gap, but corresponding data from pig or human are lacking. On the other hand, human primary nociceptors differ from rodents concerning a broader overlap between Ret and TrkA and Na_V_1.8 expression (Rostock et al., 2018), and also in their lack of correlation between small size DRG and nociceptive phenotype (Zhang et al., 2017). Thus, direct translation between species is problematic. We made use of a reporter mouse line that allows the identification of mechano-insensitive nociceptors in murine DRG cultures to compare action potential characteristics between silent nociceptors and presumed polymodal nociceptors identified by the absence of IB4-labeling and sensitivity to the TRPV1 agonist capsaicin. However, it needs to be pointed out that murine silent nociceptors are mainly innervating viscera and deep somatic tissue, but not skin (Prato et al., 2017) precluding direct translation. High levels of Na_V_1.8 were found in CHRNA3+ neurons, with their TTX-s currents being lower and the TTX-r currents higher than in presumed polymodal (IB4-/capsaicin+) nociceptors (Schaefer et al., 2018). In addition, TTX-r currents in the silent nociceptors were activated at less depolarized membrane potentials (Schaefer et al., 2018). In support of our data, higher SCNA10 and CHRNA3 expression was found in a population of isolectin B4 negative vs. IB4-positive sensory neurons among presumed nociceptors (SNS-Cre/TdTomato reporter mice; Chiu et al., 2014). However, lower SCNA10 expression was found in populations of peptidergic (CHRNA3+) vs. mas-related G protein-coupled receptor D (MrgprD)-positive neurons (Zheng et al., 2019). On a single cell level, CHRNA3 expression was found mainly within the peptidergic nociceptor population coinciding with high SCNA10 expression (Usoskin et al., 2015). Increased amplitude and duration of APs in silent nociceptors found in our study are in line with the higher functional expression of Na_V_1.8 in the silent nociceptors in mice. Thus, albeit increased Na_V_1.8 expression in murine CHRNA3+ nociceptors could be interpreted as a common axonal feature of silent nociceptors, the differential innervation pattern between species limits translatability. However, our results substantiate nociceptor-class specific differences in action potential generation and modulation also in rodents.

In summary, our data have shown that silent nociceptors differ in their axonal characteristics from polymodal nociceptors, both in pig and mouse. However, translation between species and experimental systems is not straight forward: outgrowing neurites *in vitro* cannot be simply phenotyped based on nociceptor classes found *in vivo*. Moreover, axonal calcium transients *in vitro* do not directly translate into activity-dependent slowing patterns. Yet, higher calcium transients in TTX-r axons of nociceptors in combination with the *in vivo* results of divergent TTX-sensitivity of polymodal nociceptors vs. cold and silent nociceptors on the one hand and the electrophysiological differences between identified CHRNA3+ silent nociceptors and IB4 negative polymodal nociceptors in the mouse, on the other hand, extend the concept that functional nociceptor classes are characterized not only by their sensory capacity but also by corresponding axonal characteristics that are required for adequate encoding of different noxious stimuli as hypothesized before Gee et al. (1996) and Belmonte and Viana (2008). Given, those silent nociceptors have a crucial role in the generation of ongoing pain in chronic pain patients (Kleggetveit et al., 2012; Serra et al., 2015) it will be of major interest to further specify the particular molecular setup of this fiber class that determines its excitability. Based on recent success in linking fiber class-specific expression pattern and firing behavior (Chiu et al., 2014; Zheng et al., 2019) this appears realistic and will not only improve our mechanistic knowledge of peripheral nociception, but might also provide new specific targets for analgesic treatment.

## Data Availability Statement

The datasets generated for this study are available on request to the corresponding author.

## Ethics Statement

The animal study was reviewed and approved by Regierungspräsidium Karlsruhe, Germany.

## Author Contributions

All authors have critically revised the manuscript for important intellectual content and contributed with analysis or interpretation of the work. RJ, VP, SL, OO, AK, MP, GG, RC and MS provided substantial contributions to the conception and design of the work. RJ, VP, SL, OO, FW, RR, AK, MP and RC were instrumental for data acquisition.

## Conflict of Interest

The authors declare that the research was conducted in the absence of any commercial or financial relationships that could be construed as a potential conflict of interest.

## Footnotes

^1^www.dapsys.net

## Supplementary Material

TABLE S1 Summary of differences in axonal calcium responses after action potential conduction between tetrodotoxin-sensitive (TTX-s) and tetrodotoxin-resistant (TTX-r) neurons. Under control conditions, the calcium transients per single action potential in TTX-s neurons are about 50% compared to TTX-r neurons (upper left columns). After TTX treatment TTX-r neurons remain responsive, but calcium transients per action potential are reduced by about 50% (upper right columns). When normalizing the response to electrical stimulation at 5, 10 and 20 Hz (1 s), both peak and area under the curve (AUC) do not differ between TTX-s and TTX-r neurons (lower left columns) but are reduced after TTX in TTX-r neurons (lower right columns). Statistically significant differences are marked with bold print (*p* < 0.01; *t*-tests).
