# Supplementary material for: TTX-Resistant Sodium Channels Functionally Separate Silent From Polymodal C-nociceptors
Source: Front Cell Neurosci. 2020 Feb 7;14:13. doi: 10.3389/fncel.2020.00013 (PMC7018684; doi:10.3389/fncel.2020.00013)
Supplement: TABLE S1 — Summary of differences in axonal calcium responses after action potential conduction between tetrodotoxin-sensitive (TTX-s) and tetrodotoxin-resistant (TTX-r) neurons. Under control conditions, the calcium transients per single action potential in TTX-s neurons are about 50% compared to TTX-r neurons (upper left columns). After TTX treatment TTX-r neurons remain responsive, but calcium transients per action potential are reduced by about 50% (upper right columns). When normalizing the response to electrical stimulation at 5, 10 and 20 Hz (1 s), both peak and area under the curve (AUC) do not differ between TTX-s and TTX-r neurons (lower left columns) but are reduced after TTX in TTX-r neurons (lower right columns). Statistically significant differences are marked with bold print (p < 0.01; t-tests). [file Table_1.docx]

Table S1

|  |  | Control conditions | | TTX effect in TTX-r axons | |
| --- | --- | --- | --- | --- | --- |
|  |  | TTX-sensitive (n=17) | TTX-resistant (n=26) | before  (n=12) | after  (n=12) |
| calcium transient  per **single** AP, ΔF/F0 | peak | 0.011 ± 0.004 | **0.023 ± 0.014** | 0.027 ± 0.011 | **0.013 ± 0.004** |
|  | AUC | 0.43 ± 0.32 | **1.23 ± 0.91** | 1.52 ± 0.75 | **0.51 ± 0.30** |
|  |  |  |  |  |  |
| peak calcium transient  per 1 s stimulation (normalized to single AP) | 5 Hz | 2.50 ± 0.83 | 1.92 ± 0.83 | 3.00 ± 1.08 | **1.12 ± 1.01** |
|  | 10 Hz | 3.29 ± 1.08 | 2.77 ± 1.35 | 4.06 ± 1.77 | **1.23 ± 1.03** |
|  | 20 Hz | 3.65 ± 1.58 | 3.41 ± 2.15 | 4.00 ± 2.13 | **1.20 ± 1.04** |
|  |  |  |  |  |  |
| AUC calcium transient  per 1 s stimulation  (normalized to single AP) | 5 Hz | 2.34 ± 0.83 | 1.95 ± 1.21 | 2.36 ± 0.69 | **0.91 ± 0.94** |
|  | 10 Hz | 3.40 ± 1.21 | 3.12 ± 1.85 | 3.35 ± 1.15 | **1.05 ± 0.99** |
|  | 20 Hz | 4.23 ± 2.47 | 3.84 ± 3.00 | 3.64 ± 1.75 | **0.98 ± 0.98** |
